# Impact of climate-induced floods and typhoons on geriatric disabling health among older Chinese and Filipinos: a cross-country systematic review

**DOI:** 10.1186/s12877-024-04855-z

**Published:** 2024-04-05

**Authors:** Joseph Kimuli Balikuddembe, Yafang Zheng, Don Eliseo Lucero Prisno, Robert Stodden

**Affiliations:** 1 Institute for Disaster Management and Reconstruction, Sichuan University and Hong Kong Polytechnic University, No. 122, Huanghe Middle Road Section 1, Shuangliu District, Chengdu, Sichuan China; 2 Center On Disability Studies (CDS), College of Education, University of Hawaii, Hawaii, USA; 3 Biliran Province State University, Naval Leyte, Philippines; 4 Faculty of Management and Development Studies, University of the Philippines Open University, Los Baños, Laguna, Philippines; 5 Department of Global Health and Development, London School of Hygiene and Tropical Medicine, London, UK

**Keywords:** Climate change, Flood, Typhoon, Geriatric, Disability, Health, China, Philippines

## Abstract

**Background:**

Apart from both China and the Philippines continuing to be exposed to and affected by different climate-induced hazards, in particular floods and typhoons, they are also reported to be witnessing rapid ageing populations of 60 years and older. As such, this systematic review synthesized the existing evidence about the impacts aggravated by floods and typhoons on the geriatric disabling health of older Chinese and Filipinos, respectively.

**Methods:**

Four (4) electronic databases were systematically searched to identify eligible studies published between 2000 and early 2023. This process had to confirm the Preferred Reporting Items for Systematic Reviews and Meta-Analyses guidelines (PRISMA), as well as the standard protocol registered with PROSPERO (CRD42023420549).

**Results:**

Out of 317 and 216 initial records retrieved for China and the Philippines, respectively, 27 (China) and 25 (Philippines) studies were eligible for final review. The disabling conditions they reported to affect the health of older adults were grouped into 4 categories: cognitive and intellectual, physical, chronic and terminal illnesses, and mental and psychological, with the latter identified as the most prevalent condition to affect older Chinese and Filipinos. On a sub-category level, posttraumatic stress disorder (PTSD) was the most common condition reported in 27 flood-related studies in China, while injuries and wounds prevailed in the Philippines, according to 25 typhoon-related studies.

**Conclusion:**

The increasing occurrence of extreme climate hazards, especially floods and typhoons in China and the Philippines, respectively, impacted the health of their older adults with various disabling effects or conditions. Therefore, this calls for appropriate geriatric-informed interventions in the context of climate change and rapidly ageing settings beyond China and the Philippines to others that are also prone to floods and typhoons.

**Supplementary Information:**

The online version contains supplementary material available at 10.1186/s12877-024-04855-z.

## Introduction

The Intergovernmental Panel on Climate Change (IPCC) previously reported how the global averaged surface temperatures increased over the past decades of the 20th century by 0.6 ± 0.2 °C [1]. Among other reasons, this was attributed to the dangerous anthropogenic interferences, which contribute to the rising temperatures and sea levels and, in turn, exacerbate the frequency and magnitude of climate extreme events like floods, typhoons, droughts, storms, heatwaves, landslides, wildfires, and pests and insects [2–4]. The impacts of these events are expected to disproportionately affect low- and middle-income developing countries (LMICs), especially those located in the global south, with limited financial, technical, and institutional capacity to adequately respond, cope with, resist, and recover from their impacts [1, 4, 5].

Being close to the Pacific Ocean, the South China Sea, and the Mediterranean-Himalayan belts [6–8] characterized by climatic, geophysical, and meteorological features, make China and the Philippines hotspots of frequent natural disasters [3, 8, 9]. In this regard, China was revealed to be susceptible to severe floods in Asia, with two-thirds of its territory at high risk of floods that have been increasingly happening every year between 1990 and 2021 [10–12]. On the other hand, typhoons remain the deadliest and costliest type of disaster in the past three decades to ravage the Philippines since 1991, according to the Philippine Atmospheric, Geophysical, and Astronomical Services Administration (PAGASA) [13]. Like most disasters, floods or typhoons disproportionately affect the health in terms of physical, mental, and psychological functioning and wellbeing of the world’s poorest and most vulnerable groups, like older adults, persons with disabilities, or persons with pre-existing chronic health conditions [14–18]. This, among other reasons, is attributable to the barriers they face in accessing essential health and social care and financial support, as well as the overlooking of their needs in prehospital and hospital-based emergency preparedness and disaster response plans [18, 19].

As extreme climate events, limited not only to floods and typhoons, are increasingly taking a toll and are also predicted to pose grave consequences on the global community, especially LMICs in the global south [1, 4], what will then be their fate on different vulnerable groups of the populations in China and the Philippines? This is particularly so with the older adults, as aforementioned, who are not only disproportionately affected but are also at high risk of experiencing diseases, injuries, disabling health conditions, and, above all, premature deaths during and in the aftermath of disasters or emergency crises [18, 19]. Despite the expanding body of literature on the vulnerability of older people to disasters, to date, no study has laboured to analyze how climatic and socioeconomic dynamics, which are coincidentally occurring and aggravating the disabling conditions or impacts on older persons in one or two countries. In this case, as both China and the Philippines continue to be concurrently exposed to different climate-induced hazards since the recent past, they are also among the countries reported have considerable ageing populations of over 60 years [20]. Therefore, a cross-country systematic review was conducted to synthesize the existing evidence about the impacts or disabling conditions aggravated by floods and typhoons in China and the Philippines, respectively, on the geriatric health of older people in the face of climate change. The study findings are anticipated to help inform identifying some of the viable interventions for enhancing their health, self-care, physical functioning, quality of life, wellbeing, and adaptive capacities beyond China and the Philippines, especially at this juncture when climate change and its adverse extreme events are seriously ravaging the globe.

## Methods

### Study design

This study is based on a systematic review of the literature and required no ethics approvals apart from conforming to the Preferred Reporting Items for Systematic Reviews and Meta-Analyses guidelines (PRISMA) [21]. We opted to use a systematic review since it rigorous and transparent method for comprehensively identifying and synthesizing relevant literature based on predefined protocol and eligibility criteria in order to answer a specific research question, and ultimately ensure that the final results are trustworthy. The study is part of a larger research project exploring the life experiences of persons aged 60 years and older with disabilities exacerbated by the conditions or impacts of climate-induced disasters in China and the Philippines.

### Study protocol

All steps in this review followed a standard protocol registered with the International Prospective Register of Systematic Reviews (PROSPERO) (identifier CRD42023420549).

### Inclusion and exclusion criteria

Studies were considered eligible for this systematic review if they reported or included the participants or populations of 60 years and older (Chinese and Filipinos) from China Mainland and the Philippines within their sample; synthesized different disabling conditions or impacts on older Chinese and Filipinos based on the International Classification of Functioning, Disability, and Health (ICF) of the World Health Organization (WHO) [22], in the contexts of floods and typhoons in China and the Philippines, respectively; and were published only in English from January 2000 through February 2023. Also, studies eligible for screening had to report any disabling outcomes or effects on older people or participants with validated and primary methodological designs. No specific restrictions were imposed on the sample sizes of studies considered eligible. Studies that were not in tandem with this criterion and those published as abstracts, editorials, letters, reviews, or reports were ineligible for consideration. It should be noted that a threshold of 60 years of age and above was set for participants in eligible studies in either country, despite the lack of an international standardized cutoff of years for who is an older adult or aged person, as well as variations in their defined age across different countries.

### Data sources and searches

This systematic review followed a predefined search strategy developed a priori through iterative consultations among authors who possess considerable knowledge and experience in conducting systematic reviews. In this case, relevant studies meeting the above inclusion criteria were searched and extracted from four electronic databases, including MEDLINE (via PubMed), Science Direct, Web of Science, and Google Scholar, between March and April 2023, using the Boolean-free and interchangeable search terms. The search terms combined both Medical Subject Heading (MESH) and non-MESH keywords, which were framed based on the main themes of this study (climate change, floods, typhoons, ageing, and disability) and its settings (China and the Philippines). The keywords were composed of the following search string: “climate change” OR “climate hazard” OR “climate disaster” OR “flood” OR “flooding” OR “typhoon”) AND (“older person” OR “old adult” OR “aged person” OR “seniors”) AND (“disabled” OR “person with disability” OR “person with physical disability” OR “stroke” OR “mental disorder” OR “stroke” OR “visual impairment”) AND (“vulnerable people”) AND (“health impact” OR “chronic condition” OR “non-communicable condition” OR “morbidity” or “mortality” OR “illness” OR mental health” OR “mental disorder” OR “PTSD” OR “depression” OR “stress” OR “anxiety” OR “trauma” OR “health*”) AND (“China” OR “China Mainland” OR “Chinese” OR “Philippine” OR “Filipino”). The “explode” option was used to increase the depth of the search.

### Data extraction, synthesis, and analysis

Based on the above search string, each country was searched independently in 4 databases as a subject heading, with the title or abstract, and using a combination of corresponding and interchangeable keywords. To avoid missing out eligible studies, a hand search of selective bibliographies or cited references of retrieved articles was performed to identify more relevant or additional articles. Two authors (JKB and ZY) independently screened each title, abstract, and keyword to determine their eligibility before the full text was retrieved and reviewed. The Endnote software X9 (version 20) was used to remove any duplicates and assess the quality of eligible studies retrieved based on 4- and 5-star ratings. This, for example, considered if a study had a clear aim, design, target population, design, and outcomes. Any disagreements between JKB and ZY concerning the study’s eligibility were resolved by consensus or by seeking the opinion(s) of the other authors. Meta-analysis was not possible due to the ambiguity of sample sizes and the design heterogeneity of the retrieved studies. Instead, the narrative synthesis was done to summarize the results based on authorship and publication year, title, study purpose, method or design, disaster (flood or typhoon event), participant sample for the older adults, and key finding(s) or outcome(s) on the disabling condition(s) reported in the study, as presented in Tables 1 and 2 (Additional Files 1 and 2).

## Results

### Search result

**Fig. 1:**
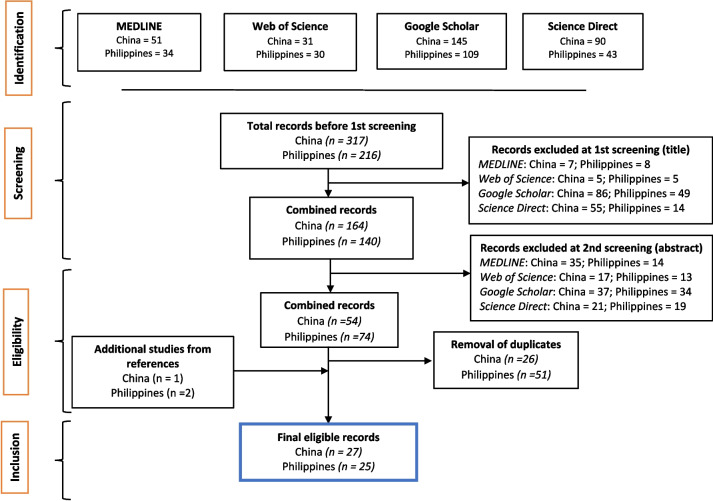
A PRISMA flow diagram for identification and identification of studies included in this systematic review

A search of four databases initially yielded 317 and 216 records for China and the Philippines, respectively. Out of these records, 150 (China) and 76 (Philippines) were excluded at the 1st screening based on their titles, thus retaining 164 and 140 for China and the Philippines, respectively. In the 2nd screening, which involved reviewing the study abstracts, 110 (China) and 66 (Philippines) records were foregone. This, brought the number to 54 and 74 studies for China and the Philippines, respectively, that were eligible for the full-text screening and review in line with the study question and objective. At this stage, the duplicates among studies were removed using the Endnote software, whereas other potential studies were identified for retrieval from their bibliographies or cited references. In the end, 27 and 25 studies for China and the Philippines, respectively, were identified as eligible for final review. Based on Endnote 4- and 5-star ratings, the overall study quality was between moderate and high and is therefore reliable to inform the discussions herein. Figure 1 presents the search results based on a PRISMA flow diagram.

### Study characteristics

Among the final eligible studies for China and the Philippines, 4 and 6 of them were respectively published in 2014 and 2016. Out of 27 studies for China, a majority of them (15) focused on the 1998–1999 Dongting Lake floods in the Hunan province [23–37]. In the Philippines, on the other hand, the largest number of studies (22 out of 25) concentrated exclusively on the 2013 super Haiyan (Yoland) typhoon [7, 16, 17, 38–56]. The remaining three studies focused on typhoons Odette [57], Ondoy-Ateneovill [58], and Vamco and Goni [59] as well. In either country, both qualitative and quantitative methods were used by the eligible studies to achieve their objectives, and they predominantly relied on cross-sectional vis-à-vis mixed methods designs (*17* in China [8, 14, 25–34, 52, 60–64] and *11* in the Philippines [16, 17, 38, 41, 44, 47, 50, 52, 57–59]). Aside from cross-sectional designs, other studies in either country were tailored to designs, including phenomenological hermeneutical approach [53, 56], ethnography [45], grounded theory [39], exploratory and content analyses [7, 15], case study analysis [38, 46], review of data or records [40, 42, 43, 48, 54, 55, 65]; retrospective design [24, 36, 49, 51], models e.g., the synthetic evaluation method (SEM) [37] and Poisson generalized linear model [66]), etc.

In general, the sample sizes of male and female participants (including the target population of older adults >  = 60 years) reported in the studies varied widely, from 10 to 75,033 in China and 6 to 6,590 in the Philippines. Apart from 9 and 7 studies on floods and typhoons for China [14, 15, 24–28, 31, 67] and the Philippines [14, 15, 24–28, 31, 67], respectively, the remaining ones did not specify the exact number of older persons (> = 60) within their sample size. The highest number of older adults in the sample sizes of flood and typhoon-related studies was 2,914 [24] and 2,020 [50] persons for China and the Philippines, respectively. In most studies, the ages of the older adults in either disaster were inconsistently reported and not clear. Participants in eligible studies were from both urban and rural settings, including residential homes and hospitals that were affected by floods and typhoons in either country (e.g., Hunan, Anhui, Henan, and Sichuan in China, while Leyte and eastern Samar provinces, the eastern Visayas region, Infanta, Quezon, and New Bataan cities, Ormoc district, etc., in the Philippines). Tables 1 and 2 (Additional files 1 and 2) show some of the characteristics of the included studies.

### Disabling conditions induced by floods and typhoons

**Fig. 2:**
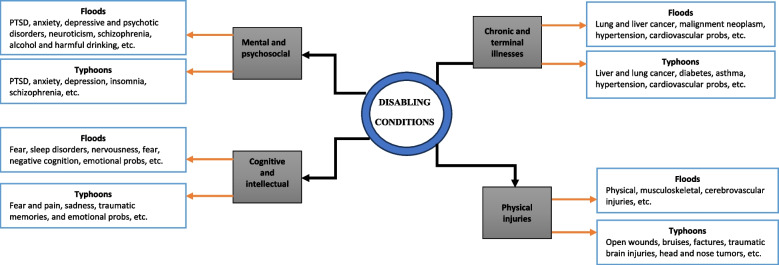
Disabling conditions associated with floods and typhoons in China and the Philippines, respectively

Participants in studies in either disaster were revealed to have witnessed and presented one or multiple short- and long-term disabling conditions (Fig. 2 and Additional file 1: Table 1). Posttraumatic stress disorder (PTSD) was the most frequent condition reported in a majority of flood-related studies in China (*n* = *16*) [23–28, 30–34, 37, 61–65, 67], while in the Philippines, physical injuries and wounds aggravated by typhoons dominated (*n* = *13*) [7, 17, 40–43, 47, 48, 51, 53–55, 58]. In China, PTSD was followed by anxiety (*n* = *9*) [23, 27, 28, 61–65, 67], depression (*n* = 9) [23, 28, 60–65, 67], physical injuries and wounds (*n* = *6*) [14, 29, 31, 36, 37, 68], and chronic and terminal illnesses (*n* = *3*) [8, 14, 29]. In the Philippines, on the other hand, injuries and wounds were followed by emotional problems (*n* = *9)* [7, 17, 38, 39, 45, 56–59], PTSD (*n* = *6*) [17, 41, 43, 46, 49, 52], chronic and terminal illnesses (*n* = *5*) [42, 43, 48, 51, 54], and trauma (*n* = *5*) [38, 39, 49, 50, 54]. Other disabling conditions, including schizophrenia [66], neuroticism [62], insomnia and sleep disorders [14, 52], and harmful alcohol drinking [44], were also divulged as having been associated with floods and typhoons in China and the Philippines, respectively. Overall, a combination of these disabling problems was grouped into four categories: cognitive and intellectual disabilities, physical, chronic and terminal illnesses, and mental and psychological conditions. The latter was the most prevalent condition in nearly two-thirds of flood and typhoon-related studies. A summary of the conditions and their subgroups is presented in Fig. 2 and Tables 1 and 2 (Additional files 1 and 2).

## Discussions

This study synthesized the existing evidence about the disabling conditions among older persons that are aggravated by floods and typhoons, respectively, in China and the Philippines —the two countries that are witnessing a rapid ageing population [20, 69], as well as remaining highly vulnerable to extreme climate events in Asia–Pacific [4, 5]. Various studies have reported climate change and its epiphenomena, including but not limited to floods and typhoons, that may not only cause mortality, morbidity, injuries, and physical damages but also heighten the risk of post-disaster public health problems [19, 29, 54]. This impacts survivors, especially those who are considered vulnerable groups, like older adults. In particular, both floods and typhoons have been pointed out in this systematic review as having direct and indirect effects on the physical, social, mental, psychological, cognitive, and intellectual functioning of older persons. In this case, whenever floods and typhoons, which are among the most frequent and deadliest natural disasters in China and the Philippines [3, 5, 10, 13] occur, their people age 60 or older —who make up about 11.9% and 5.1% of the population, respectively [69], are at the highest risk of experiencing a wide range of disabling health conditions they induce compared to younger age groups.

Older persons tend to experience negative mental, psychiatric, or psychological outcomes, perhaps more than any other health-related effects in the aftermath of disasters. Among the outcomes is PTSD, which is the most prevalent disabling condition witnessed. This has not only been corroborated by the findings herein, but PTSD was also previously named as the most significant type of psychiatric morbidity in older people after disasters such as earthquake and tsunami [70, 71]. Moreover, one systematic review found that older adults were 2.11 times more likely to experience PTSD symptoms and 1.73 times more likely to develop adjustment disorder when exposed to natural disasters as compared to younger adults [72]. Apart from age, fragility, senility, and social support, functional, mental, or cognitive limitations can be attributed to the predictors of a high incidence of PTSD among older adults in times of floods, typhoons, or other disasters. Oftentimes, PTSD can manifest in symptoms of depression, anxiety, stress, trauma, psychotic disorders, or neuroticism, which symptoms this systematic review revealed to have been previously induced by floods and typhoons among older Chinese and Filipinos, respectively. On top of that, PTSD is further worsened by traumatic feelings, negative emotions, thoughts, and feelings, nervousness, fear, pain, insomnia or sleep-related problems, and harmful alcoholic drinking [7, 15, 38, 41, 56, 58, 60, 73], as well as discrimination, social prejudice, isolation, and racism [45] as also disclosed by this systematic review.

For long, the above conditions have been flagged as post-flood or typhoon effects with detrimental mental and psychological impacts and memories on older adults [73]. This, for instance, can happen after they lose their loved ones, homes, income, and property; are separated or cut off from their family, friends, and social networks; are displaced and evacuated to a new and unfamiliar environment; sustain injuries and other health-related complications; or lose special, preferred foods, diets, or daily medications [74]. These challenges were reported to exacerbate not only the symptoms of mental illnesses as highlighted above among the older adult survivors but also impair their functional ability, well-being, activities of daily living (ADL), and quality of life (QoL). These challenges, for example, were reported to have been associated with the 1998–1999 floods in Hunan Province, China [23–37], and the 2013 Haiyan typhoon in the Philippines [7, 16, 17, 38–56] —the predominant climate-induced disasters revealed to have struck the two Asian countries by this systematic review. In addition, older adults are made more vulnerable to effects of disasters beyond floods and typhoons because of their preexisting or concurrent psychiatric comorbidities, including disabilities, cognitive decline, limited social networks, and socio-economic changes [50]. These conditions may end up imposing mobility restrictions and also constraining them from perceiving and responding to emergency warnings.

Faced with not only the mental or psychological effects of floods and typhoons —whether during or in their aftermath, ageing people were also exposed to life-threatening injuries, traumatic disorders, and chronic and terminal illnesses. This systematic review has revealed varying physical injuries, which emanated from musculoskeletal wounds, bruises, lacerations, factures, and head and ear tumors, etc.) [31, 40, 42, 43, 48, 51] to have predominantly affected the Filipino older adult compared to Chinese, as well as chronic or terminal conditions (e.g., cancer, cardiovascular diseases, hypertension, diabetes, asthma, etc.,) [8, 29, 42, 48, 51]. The high prevalence of physical injuries in Filipino older adults could been due to the heavy and circulating masses of winds and thunderstorms, which could have not only destroyed the homes, property, and other infrastructure they were occupying and using, but also injured them when the typhoons struck. This, in turn, affects their performance of ADL (e.g., bathing, dressing, eating, moving around, and using the bathroom) and, in general, their health-related QoL [74]. One review article that examined the relationship between disaster preparedness and chronic diseases among older adults indicated that about 80% of them at least suffer from one chronic condition. As a result, this aggravates their vulnerability to different problems or conditions of disasters [75], which subsequently undermines their ability to adequately prepare, respond, recover, or cope with the short- and long-term health impacts of floods, typhoons, or other disasters.

Lastly, this review has certain limitations. Since only English studies were considered, this is likely to have not only accrued language bias but also excluded some eligible studies, especially those published in Chinese and Filipino languages. In addition, the review timeline between January 2000 and February 2023 could have contributed to the exclusion of other flood and typhoon events, making eligible studies to majorly focus on the 1998–1999 Dongting Lake floods [23–37] and 2013 super typhoon Haiyan [7, 16, 17, 38–56] in China and the Philippines, respectively. Also, Tables 1 and 2 (Additional files 1 and 2) demonstrate substantial heterogeneity among the studies with differing characteristics, which was problematic to pool for meta-analysis. Nevertheless, some insights are herein offered about the disabling conditions associated with floods and typhoons, upon which appropriate interventions to address them should be taken, especially those aimed at enhancing the health, self-care, physical functioning, QoL, wellbeing, and adaptive capacities of older persons in the settings witnessing rapid ageing populations.

## Conclusions

As climate change progresses, some of its extreme hazards, in particular floods and typhoons, will also increase in frequency and severity due to rising sea levels and disproportionately affect the most vulnerable groups, such as the older persons. As such, this systematic review endeavored to explore the linkages between climate change, ageing, and disability. Accordingly, over the past years, this review found floods and typhoons to have aggravated various disabling conditions among Chinese and Filipino older adults, respectively. As the ageing population is rapidly rising amid the increasing extreme climate events beyond floods and typhoons in China and the Philippines, respectively, appropriate geriatric-based responses need to be prioritized in both countries. This especially ought to consider interventions aimed at addressing the risk and impacts of mental, psychological, physical, chronic, cognitive, and intellectual disabling conditions faced by older adults due to floods and typhoons, as revealed by this systematic review. This, however, ought to be augmented by further research focusing not only on China and the Philippines but also on other flood- and typhoon-prone settings.

### Supplementary Information

**Supplementary Material 1.**

**Supplementary Material 2.**

## Data Availability

The datasets used and/or analysed during the current study available from the corresponding author on reasonable request.

## Abbreviations

IPCC: Intergovernmental Panel on Climate Change
PAGASA: Philippine Atmospheric, Geophysical, and Astronomical Services Administration
PRISMA: Preferred Reporting Items for Systematic Reviews and Meta-Analyses guidelines
PROSPERO: Prospective Register of Systematic Reviews
PTSD: Posttraumatic stress disorder
ICF: International Classification of Functioning, Disability, and Health
