# Supplementary material for: Impact of climate-induced floods and typhoons on geriatric disabling health among older Chinese and Filipinos: a cross-country systematic review
Source: BMC Geriatr. 2024 Apr 5;24:320. doi: 10.1186/s12877-024-04855-z (PMC10998398; doi:10.1186/s12877-024-04855-z)
Supplement: Supplementary file 1 — Supplementary Material 1. [file 12877_2024_4855_MOESM1_ESM.docx]

**Table 1.** Characteristics of flood-related studies in China

| **Author (Year)** | **Study purpose** | **Study method (design)** | **Flood event (setting affected)** | **No. elderly participants** | **Disabling condition(s)** | **Summarized key outcome(s)** |
| --- | --- | --- | --- | --- | --- | --- |
| Wu J, et.al. (2015)**^[14]^** | To investigate the health status and the HRQoL of the elderly in Bazhong city after a major flood in 2011. | Nationwide standardized survey | Bazhong flood in Sichuan province | 1,200 elderly people Ages (60-79 =88.2%; 80-99 =11.8%) | Poor physical health, poor sleep patterns, and chronic diseases, etc. | There was a marked decline in health status among elderly in Bazhong after the 2011 flood. |
| Liu A, et.al. (2008)**^[23]^** | To construct a short screening scale for PTSD. | Multistage sampling method | 1998 and 1999 floods in Hunan province | Unspecified out of 27,267 participants (No ages) | Risk of anxiety, depression, and PTSD | The 7-symptom screening scale developed in this study is highly valid, reliable, and predictable, and is an effective tool to screen quickly for PTSD in large epidemiologic studies among victims of natural disasters, e.g., floods. |
| Liu A, et.al. (2006)**^[24]^** | To estimate the occurrence and to assess the determinants of PTSD in flood victims. | A retrospective study involving face-to-face preconstructed questionnaire interviews. | 1998 and 1999 floods in Hunan province | 2,914 subjects >60 out of 33,340 total participants | PTSDs occurred in about 8% to 9% of flood victims and their risk increased in female and older victims | PTSD is a common mental disorder in flood victims, and calls for improved mental health services, especially for female victims and senior victims in the flood-affected areas. |
| Zhong S, et.al. (2020)**^[67]^** | To evaluate the effectiveness and pathways of planned shelters in protecting mental health of flood victims in China. | A two-phase field investigation | 2016 severe flash floods in Anhui Province | >60 years old 152 (45.0%) out of 675 respondents | Anxiety, depression, and PTSD | Planned sheltering cannot only be used to achieve better mental health outcomes in China but also inform other flood-prone areas to mitigate psychological vulnerability of IDPs. |
| Wei Q, et.al. (2020)**^[66]^** | To quantify the relationship between floods and admissions for schizophrenia in Hefei, China. | A Poisson generalized linear model combined with a distributed lag non-linear model | 2005 - 2014 floods in Hefei, Anhui province | Unspecified out of 36,607 cases of schizophrenia | Schizophrenia | There was a significant association between floods and admissions risk for schizophrenia. |
| Dai W, et.al. (2016)**^[25]^** | To explore the prevalence rate of current PTSD and examine the association between social support and recovery prior PTSD after the 1998 flood of Dongting Lake. | Cross-sectional follow-up survey (e.g., using face-to-face interviews and structured questionnaire) | 1998 Dongting Lake floods in Hunan province | 114 participants aged ≥60 out of 321 subjects | PTSD | Prevalence rate of current PTSD indicates that natural disasters e.g., floods, may affect the mental health of victims for a long time. |
| Dai W, et.al. (2017)**^[26]^** | To explore the association between brain-derived neurotropic factor Val66Met polymorphism and PTSD among flood survivors in China. | Cross-sectional (e.g., face-to-face interviews using PTSD Checklist-Civilian version and DNA for genotyping) | 1998 Dongting Lake floods in Hunan province | 72 (41.1%) participants aged ≥60 out of 175 total subjects | Prevalence of PTSD | Met carriers for BDNF rs6265 are at higher risk of developing PTSD and also exhibiting more severe PTSD symptoms than Val/Val homozygotes among flood survivors in China |
| Dai W, et.al. (2017)**^[27]^** | To estimate the extent to which PTSD and anxiety co-occur in flood survivors, and identify shared risk factors for PTSD only and comorbidity of PTSD and anxiety. | Cross-sectional study | 1998 Dongting Lake floods in Hunan province | 146 elderly (44.9%) aged 60-87 out of 325 participants | PTSD and anxiety | Comorbidity of PTSD and anxiety are prevalent in flood survivors due to the intensity of flood exposure and personality traits. |
| Tan and Schultz (2021)**^[68]^** | To provide a feasible way to obtain on-site damage information prior to conducting comprehensive surveys in disaster-stricken areas for real-time managing strategies for regional flood mitigation and managing sustainable development of flood-prone areas. | Social-media big data (specifically, Weibo) | August floods 2020 in Chongqing | Unspecified out of 25,811 total texts from social media users | Negative emotions | In the Chongqing flood, the local residents tended to express more negative emotions. |
| Qin Y, et.al. (2023)**^[8]^** | To examine the disaster perception and preparedness of residents in Shenzhen, China and also measure the awareness of residents with and without chronic disease on the types of disasters that might occur in Shenzhen, the sources of information considered useful in preparing for disasters, and how individuals and families prepare to deal with disasters. | Cross-sectional study based on large community survey | Floods in Shenzhen coastal city | Unspecified out of 2,421 residents | Chronic diseases (e.g., diabetes, cancer, or end-stage renal disease) | Although participants with chronic diseases were better prepared than those without, overall, the Shenzhen residents were inadequately prepared for disasters and needed public education. |
| Zhen R, et.al. (2018)**^[60]^** | To examine the prerequisites of sleep problems amongst a traumatized population and assess the underlying mechanism of sleep problems following trauma. | Cross-sectional design (e.g., qualitative questionnaires) | July 2016 flood in Wuhu city, Anhui province | Unspecified out of 187 flood victims | Sleep problems, fear, negative cognition, and depression | Sleep problems of flood victims were elicited by the combined role of fear, negative cognition, and depression following trauma. |
| Liang Y, et.al. (2023)**^[61]^** | To identify the latent profiles of psychological status and acceptance of change among Henan residents who have been cumulatively exposed to these floods and the COVID-19. | Cross-sectional based on Latent profile analysis (LPA) | July 2021 rain-triggered floods in Henan Province | Unspecified out of 977 participants | Post-traumatic stress symptoms (PTSS) and anxiety | Heterogeneous psychological responses among residents cumulatively exposed them to floods and COVID-19 pandemic. |
| Dai W, et.al. (2017)**^[28]^** | To estimate the prevalence of PTSD and anxiety among flood survivors 17 years after the 1998 Dongting Lake flood and to identify the risk factors for PTSD and anxiety. | Cross-sectional design | 1998 Dongting Lake floods in Hunan province | 146 (44.9%) aged 60–87 out of 325 total participants | Prevalence of PTSD and anxiety | PTSD and anxiety were common long-term adverse psychological outcomes among flood survivors. |
| Abuaku BK, et.al. (2009)**^[29]^** | To elucidate the role of socioeconomic status in morbidity and mortality among populations suffering floods in Hunan province. | Cross-sectional design | 1998 Dongting Lake flood in Hunan province | Unspecified out of 40,028 total cases | Road traffic injuries, lung & liver cancer, and cerebrovascular accident, etc. | Gender, age group, source of drinking water, type of flood suffered, and severity of flood played highly significant roles in morbidity. |
| Cui K and Sim T. (2017)**^[15]^** | To explore older people’s needs from a psychosocial perspective. | Exploratory qualitative descriptive research | No specific floods reported to have affected people near the epicenter of 5.12 Wenchuan earthquake. | 10 participants aged >=60 | Constant fear | Older people being exposed to disasters will not only develop psychological problems but also suffer disruption to their social support network. |
| Jing X, et.al. (2022)**^[62]^** | To analyze the impact of floods, social support, and personality on the mental health of residents in Henan Province, provide fundamental knowledges for making measuring strategies to improve the psychological protection and anti-stress ability of the residents after the disaster. | Cross-sectional based on online survey platform | July 2021 flood in Zhengzhou city, Henan province | Unspecified out of 572 total participants | PTSD and neuroticism for depression-anxiety-stress | Residents living in the catastrophic flooding areas had significant post-traumatic mental health issues. |
| Wu J, et.al. (2019)**^[65]^** | To identify the good practice and lessons learned from China's response to severe flooding in Anhui province in 2016. | Data review based on interviews and thematic analysis | 2016 flooding in Anhui Province | Unspecified out of 21 total interviewees | Distress, anxiety, depression, and PTSD | To reduce the health impacts including those affecting mental health and status caused by severe flooding, health ministries and other institutions needed to cooperate closely to establish planned mass shelters. |
| Huang P, et.al. (2010)**^[30]^** | To establish a prediction model for the occurrence of PTSD among adults in flood districts. | Cross-sectional design (i.e., face-to-face interview questionnaires) | 1998 Dongting Lake floods in Hunan province | Unspecified out of 25,478 total participants | PTSD | A simple risk score model can be used to predict PTSD among victims of flood. |
| Dai W, et.al. (2016)**^[31]^** | To measure the prevalence rate of PTSD among survivors of 1998 Dongting Lake flood who were diagnosed with PTSD in 2000 at follow-up and identify predictors of recovery from the PTSD diagnosis in 2000. | Cross-sectional follow-up study | 1998 Dongting Lake floods in Hunan province | 59 (29.4%) aged 61-88 out of 201 total survivors | PTSD and bodily injury | The prevalence rate of PTSD at follow-up indicated that natural disasters such as floods may have a negative influence on survivors’ mental health for an extended period of time. Individuals who lost relatives, suffered from bodily injury, had a low level of social support, or had a negative coping style were less likely to recover from PTSD. |
| Golitaleb M, et.al. (2022)**^[32]^** | To explore the prevalence and determinants of chronic post-traumatic stress disorder (PTSD) among flood victims. | Cross-sectional survey | 1998 floods in Hunan province | Unspecified out of 123 total individuals | Chronic PTSD and nervousness | Prevalence of PTSD was relatively high in the flood victims and required taking necessary preventive, supportive, therapeutic, and effective actions for them. |
| Liu N and Ma Z. (2021)**^[63]^** | To illuminate the underlying mechanism of how viewing short videos about the 2021 Henan floods triggers personal psychiatric reactions among a non-exposed population | Cross-sectional survey questionnaire | 2021 Henan floods | Unspecified out of 516 total respondents | Mild depression, anxiety, and PTSD severity | Viewing media content that induces emotional responses is inevitable. |
| Wang Z, et.al. (2023)**^[64]^** | To investigate the mental health status and related influencing factors of local ordinary residents after the flood, and to provide reference for government departments to formulate disaster psychological intervention countermeasures based on evidence-driven strategies. | Cross-sectional survey | July 2021 floods in Zhengzhou city, Henan province | Unspecific out of 469 total participants | Rainstorm-related stresses, PTSD, depression, and anxiety symptoms. | Rainstorm could cause local residents to have various degrees of psychological symptoms. |
| Hu S, et.al. (2015)**^[33]^** | To estimate the chronicity rate and identify the prognostic factors of PTSD in flood victims. | Cross-sectional follow-up study using community-based survey | 1998–1999 floods in Hunan province | Unspecified out of 25,478 total subjects | Probable PTSD and flood-related stressors due to life-threatening experiences, extreme physical and psychological adversity. | Demographic characteristics (e.g., age, gender, and education level) seemed to have no influence on the recovery from PTSD, and trauma-related stressors and social participation are important predictors for remission from chronic PTSD. |
| Feng S, et.al. (2007)**^[34]^** | To explore the relationship between social support and PTSD among flood victims. | Cross-sectional follow-up survey | 1998 Dongting Lake floods in Hunan province | Unspecified out of 25,478 total participants | PTSD | PTSD in flood victims is significantly associated with social support. |
| Tan HZ, et.al. (2004)**^[35]^** | To assess the impact of flood on the quality of life (QOL) of residents in the affected areas in China. | Qualitative design based on natural experiment approach | 1998 Dongting Lake floods in Hunan province | Unspecified out of 494 total respondents | Physical discomforts, motion and negative feelings, mental tension, | Flood has a negative impact on the QOL of residents in the affected areas, especially farmers, seniors, persons with introvert personalities and adverse life-events. |
| Hongzhuan T, et.al. (2006)**^[37]^** | To establish a synthetic evaluation model (SEM) that can be used to analyze flood hazards. | Retrospective study based on synthetic evaluation model (SEM) and Delphi procedure | 1998 floods in Hunan province | Unspecified out of 20,230 families and 75, 033 people | Injuries and PTSD | A SEM can be used to evaluate flood hazards, as well as to assist public health-care workers provide appropriate flood disaster management. |
| Li X, et.al. (2007)**^[36]^** | To examine the health effect of different types of floods in the Dong-Ting Lake area using years of potential life lost (YPLL). | Retrospective study based on survey interviews and questionnaires | 1998 Dongting Lake floods in Hunan province | Unspecified out of 24,659 total residents in river flood groups | Injury and malignment neoplasm | Floods increase the affected residents’ YPLL, and that the river flood had stronger effects than the drainage problems floods. |
