# Supplementary material for: Impact of climate-induced floods and typhoons on geriatric disabling health among older Chinese and Filipinos: a cross-country systematic review
Source: BMC Geriatr. 2024 Apr 5;24:320. doi: 10.1186/s12877-024-04855-z (PMC10998398; doi:10.1186/s12877-024-04855-z)
Supplement: Supplementary file 2 — Supplementary Material 2. [file 12877_2024_4855_MOESM2_ESM.docx]

**Table 2.** Characteristics of typhoon-related studies in the Philippines.

| **Author (Year)** | **Study purpose** | **Study method (design)** | **Flood event (setting affected)** | **No. elderly participants** | **Disabling condition(s)** | **Key outcome(s)** |
| --- | --- | --- | --- | --- | --- | --- |
| Albougami, et al. (2023)**^[7]^** | To explore older Filipino adults’ living experiences pertaining to disaster during Typhoon Haiyan, highlighting the problems they encounter after the disaster, and identifying factors that contribute to their disaster resilience. | Case study design | 2013 Typhoon Haiyan - Eastern Philippines | 11 participants >=60 yrs. | Isolated, sad, disappointed, pain, hurt, and negative thoughts and feelings & leg injury | Gerontologists need to suggest for older adults to consider social support activities to provide social contact, reduce alienation and isolation, and increase a sense of belonging, of meaning, and of life purpose. |
| Adviento & Guzman (2010)**^[58]^** | To examine the positive qualities, themes, and manifestations of community resilience that were reflected among the Ateneoville residents, which enabled them to survive and bounce back from the disaster caused by Typhoon Ondoy. | Cross-sectional (e.g., survey and Focus group discussion) | 2009 Typhoon Ondoy - Ateneoville | 2 out 6 participants aged >=60. | Physical and emotional discomfort, and fear | Positive psychology or strengths-based approach to psychosocial interventions for survivors of natural disasters that goes beyond understanding weaknesses and pathologies help to capitalize on strengths and virtues in view of achieving optimal human functioning in the wake of disaster adversities. |
| Almazan, et.al. (2018)**^[38^**] | To explore the disaster resiliency among older adult after a traumatic experience with the typhoon Haiyan. | Cross-sectional (e.g., Focus group discussion) | 2013 Typhoon Haiyan | 12 participants aged 60 - 68 yrs. | Traumatic and fearing experience | During a disaster, social support and culture are intertwined with each other. This contributes to resiliency among older adult and construct proactively cultivated people’s adaptation skills that can be adjusted essentially during a traumatic situation. |
| Almazan A, et.al. (2019)**^[39]^** | To understand the experiences of older adult survivors of Typhoon Haiyan before, during, and after the disaster. | Grounded theory | 2013 Typhoon Haiyan - Eastern Philippines | 14 participants aged 65 - 72 yrs. | Emotional distress and traumatic memories | Contextual factors (i.e., perception of disaster, self-regulating behaviors & positivity) played an important role in the post-disaster recovery process. |
| Marom S, et.al. (2014)**^[40]^** | To describe clinical and ethical dilemmas in patients presenting with head and neck (H&N) tumors to a field hospital in the “subacute” period following a typhoon. | Retrospective data and record review | 2013 Typhoon Haiyan (Yolanda) | Unspecified out of 85 total patients | H &N tumors, of which thyroid neoplasms were the most common | Surgical interventions in patients with advanced H&N tumors in a relief mission setting can be performed for therapeutic, palliative, and diagnostic purposes. |
| Hechanova D, et.al. (2018)**^[16]^** | To evaluate the effect of a community-based resilience intervention for Filipino displaced survivors of Super Typhoon Haiyan. | Cross-sectional (e.g., Quasi-experimental) | 2013 Typhoon Haiyan - Tacloban City | Unspecified out of 96 participants | Anxiety | Resilience intervention Katatagan (the Filipino term for strength or resilience) leads to positive outcomes on anxiety and resilience of Filipino survivors. |
| Salipong BR. (2023)**^[57]^** | To assess the experiences, coping mechanism and concern of BSAVWA (Bonbon Small Vendor and Worker Association) with super typhoon Odette in Barangay Bonbon Clarin, Bohol through focus group discussion and coping mechanism questionnaire. | Cross-sectional (e.g., FGD and survey questionnaire) | 2021 Super-typhoon Odette - Clarin coastal town | Unspecified out of 30 participants | Terrible fear | Programs and activities that help respondents on their holistic recovery from the unpleasant experience and mitigate stress in every natural disaster and improve their health and wellness need to be created. |
| Hugelius, et al. (2017)**^[41]^** | To describe survivors’ and health professionals’ health 30 months after a natural disaster and to evaluate the health effects of disaster response interventions, with a focus on disaster radio. | Cross-sectional study (i.e., using a web-based survey) | 2013 Typhoon Haiyan - Tacloban area, Leyte province | 18 of >=66 out of 443 participants | Traumatic brain injuries, pain, and psychosocial/mental health effects (e.g., PTSD, Anxiety or depression) | There were short-term and long-term physical, psychological, and social consequences for survivors as a result of the Haiyan typhoon. Mental health problems were more frequent and lasted longer than physical problems. |
| Salazar, Law, et.al. (2017)**^[42]^** | To analyze the health impact of communicable diseases, injuries, and non-communicable diseases (NCD) during Typhoon Haiyan using the SPEED database including descriptions of individual syndromes and the use of health facilities by different age groups during disaster response and recovery. | SPEED reports medical consultations | 2013 Typhoon Haiyan - Eastern Visayas region | Unspecified among the 3,425 | Open wounds and bruises/burns, fractures, high blood pressure, acute asthmatic attack, and diabetes mellitus, etc. | Interventions targeting young, elderly, and impoverished populations are recommended, and community health centers should be prioritized in their recovery and rehabilitation. |
| Chang, Simkin, et.al. (2016)**^[43]^** | To improve disaster preparedness by analyzing medical diagnoses presenting to a city district hospital before, during, and after Typhoon Haiyan. | Retrospective review of data records | 2013 Typhoon Haiyan - Ormoc District Hospital (ODH) | Unspecified out 3,600 patients of >=5 yrs. | Motor vehicle collision and musculoskeletal related injuries, laceration/stab wound, fracture, cerebral concussion/contusion, electrocution, cardiovascular, and psychiatric conditions, etc. | ODH had a significantly increased number of cases of infectious diseases, including exacerbations of chronic health conditions, etc. |
| Czaicki, Fabrigas, et.al. (2015)**^[44]^** | To determine the extent of alcohol-related problems and to train local health staff to provide community-based alcohol intervention services. | Cross-sectional (e.g., using Cut, Annoyed, Guilty, and Eye (CAGE) questionnaire and Alcohol Use Disorders Identification Test (AUDIT)) | 2013 Typhoon Haiyan -Tacloban City | Unspecified out of 239 participants of >=18 yrs. | Alcohol and harmful drinking problems | Addressing alcohol-related issues in the community is an important public health intervention. |
| Cuaton and Su (2020)**^[45]^** | To provide insights on how local indigenous knowledge and practices helped the Mamanwa indigenous peoples in the Philippines reduce their disaster risk in the onslaught of Typhoon Haiyan in 2013. | Mixed method (e.g., FDG, questionnaire, and key informant interview (KII), observation and literature review) | 2013 Typhoon Haiyan -Eastern Visayas region | Unspecified out of 12 participants of 38 - 68 yrs. | Social prejudice, discrimination and fear based on their skin color, hairstyle, physical features, indigenous language, beliefs, customs, and practices | Much as Mamanwas came a long way in terms of integrating and being accepted by locales, they were subjected to various types of discrimination that may affect their willingness and interest in sharing their local-indigenous knowledge. |
| Acosta, Eugenio, et.al. (2016)**^[46]^** | To investigate the types and scale of loss and damage in Infanta, Quezon and New Bataan that were affected by typhoon-induced floods and landslides in 2004 and 2012, and also assess people’s preferences on adaptation measures and perceptions on human-nature links on occurrence of disasters. | Case studies | 2013 Typhoon Haiyan - Infanta, Quezon and New Bataan municipalities | Unspecified out of 200 respondents | Anxiety and psychological distress | Human loss and property damage due to typhoon-induced floods and landslides caused psychological distress to affected people. |
| Aruta, Crisostomo, et.al. (2022)**^[59]^** | To examine the psychometric properties and antecedents of positive mental health of Filipinos within the context of the disasters caused by typhoons Vamco and Goni that occurred at peak of COVID-19 crisis. | Cross-sectional (using Web-based survey) | 2020 Typhoons Vamco and Goni - Quezon province, Bicol & Calabarzon regions | Unspecified out of 447 participants aged 18 - 70 yrs. | Effects on emotional, social, and psychological well-being | The findings provided meaningful and relevant insights for promoting positive mental health in communities in times of great adversities. |
| Leonardi, Talampas, et, al. (2016)**^[47]^** | To assess disability among the survivors of the Haiyan/Yolanda typhoon that struck Philippines in 2013. | Cross-sectional survey | 2013 Typhoon Haiyan/Yolanda - Eastern Philippines | Unspecified out of 1,982 survivors aged 18+ yrs. | Severe disability | Survivors living in rural contexts and those who received tools/materials to repair their houses were less likely to have higher disability. |
| Cuesta, Loenhout, et.al. (2020)**^[48]^** | To describe the reasons for medical consultation in the field hospital operated by MM (Mercy Malaysia) after typhoon Haiyan. | Observational study based on data records | 2013 Typhoon Haiyan - Ormoc City | Unspecified out of 1,425 patients aged 50+ yrs. | Injuries (e.g., open wounds, bruises, and fractures etc.) and NCDs (e.g., High blood pressure, acute asthmatic attack, diabetes mellitus, etc.) | Due to a high initial load of injuries, effective management to them is essential during the weeks following a typhoon. |
| Weintraub, Garcia, et.al. (2016)**^[49]^** | To report the monthly volume of MH activities and beneficiaries; sociodemographic and care seeking characteristics of beneficiaries receiving MH counselling/care, stratified by the severity of their condition; profile and outcomes of patients with severe mental disorders; prescribing practice of psychotropic medication; and main factors facilitating the identification and management of individuals with severe mental disorders. | Retrospective review of programme data | 2013 Typhoon Haiyan -Eastern Samar Province | Unspecified out of 134 patients aged 45+ yrs. | Anxiety disorders, post traumatic reactions, depressive and psychotic disorders, and other behavioral-related problems | The study demonstrated a need for provision of mental health care for people with severe mental disorders during emergencies, particularly those with pre-existing conditions. |
| Almazan, Cruz, et.al. (2018)**^[50]^** | To investigate older adults’ adaptation patterns after a super-typhoon in the Philippines. | Cross-sectional study | 2013 Typhoon Haiyan -Tacloban City | 2,020 participants of >=65 yrs. | Traumatic experiences | There is a need for nurse gerontologists to promote spirituality and encourage positive attitudes among older adults. |
| Mobula, Fisher, et.al. (2016)**^[51]^** | To present a post disaster evaluation of the burden of hypertension among victims of Super Typhoon Haiyan in the Philippines. | Retrospective observational study | 2013 Typhoon Haiyan - central Leyte and Western and Eastern Samar, island | Unspecified out of 3,730 adults >=18 yrs. | Hypertension (HTN), puncture wounds, and blood pressure (BP) | Evidence-based guidelines on the management of hypertension and other NCDs during humanitarian emergencies were limited. |
| Chan, Tang, et.al. (2013)**^[17]^** | To examine the psychological impact of the typhoon and its aftermath, as well as the impact of individual typhoon related stressors among a group of survivor-responders. | Cross-sectional study | 2013 Super Typhoon Haiyan | Unspecified out of 237 adult Filipinos | PTSD, physical injury, and perceived life threat | The psychological impact of Super Typhoon Haiyan on survivor-responders in terms of nonspecific psychological distress and symptoms of post-traumatic stress was considerable and were associated with the financial instability, physical injury, and perceived life threat. |
| Labarda & Chan (2018)**^[52]^** | To examine sleep disturbances and their relation with posttraumatic stress and general psychological distress after the 2013 Super Typhoon Haiyan. | Cross-sectional study (e.g., workshop training, questionnaire and data analysis) | 2013 Typhoon Haiyan - Tacloban City | Unspecified out of 361 Filipino adults | PTSD, insomnia, and general psychological distress (GPD) | Study findings underscore the longer-term relationship between sleep disturbances and overall sleep quality to posttraumatic stress in the context of a natural disaster. |
| Garcia, et.al. (2016)**^[53]^** | To explore the lived experiences of the older adults affected by the Typhoon Yolanda (Haijan). | Descriptive phenomenological interviews and FGDs | 2013 Typhoon Haiyan - Northern Cebu | 9 participants of >=60 yrs. | Physical immobility, fear, and helplessness etc. | Lived experiences of older adults were indeed a life changing process ranging from the undesirable to life giving experiences. |
| Savage, Christian, et.al. (2015)**^[54]^** | To describe the health care needs of 6,596 members of the local population treated by the medical section of the Canadian Armed Forces (CAF) Disaster Assistance Response Team (DART) during their 1-month deployment to the Philippines after Typhoon Haiyan. | Prospective data analysis | 2013 Typhoon Haiyan - Panay Island | Unspecified out of 6,590 Filipino adults | Trauma and musculoskeletal injuries, cardiovascular diagnoses, psychiatric, respiratory, and neurological conditions | Data collection ought to continue on all future missions and be modified to provide further information to larger disaster coordination teams, such as UN OCHA. |
| van Loenhout, Cuesta, et.al. (2018)**^[55]^** | To investigate the short-term impact of Typhoon Haiyan on admissions in two hospitals that remained operational during and after the typhoon in Eastern Visayas, Philippines. | Patient data analysis | 2013 Typhoon Haiyan - Eastern Visayas Regional Medical Center (EVRMC) in Tacloban, and Ormoc District Hospital (ODH) in Ormoc | Unspecified out of 6,590 Filipinos aged 50+ yrs. | Injury, diseases of respiratory system, surgery, and orthopedics | The study highlighted evidence about the higher risk of admission faced by children and elderly following an acute event, as well as particular recommendations for operational activities after a tropical cyclone. |
| Hugelius et al. (2015)**^[56]^** | To describe survivors’ experiences of being in the immediate aftermath of a natural disaster and the impact disaster radio made on recovery from the perspective of the individuals affected. | Phenomenological hermeneutical method | 2013 Typhoon Haiyan | Unspecified out of 28 informants of 18+ yrs. | Emotional pain, uncertainity, and fear | Music contributed to emotional endurance and reduced feelings of loneliness. |
